# Faculty of sports science students, physical education teachers, and athletes’ level of knowledge and attitude about mouthguards

**DOI:** 10.1186/s12903-023-03675-8

**Published:** 2024-01-09

**Authors:** Aslı Soğukpınar Önsüren, Hüseyin Eroğlu, Cemil Aksoy

**Affiliations:** 1 Department of Pediatric Dentistry, Faculty of Dentistry, Mersin University, Mersin, Turkey; 2 Faculty of Sports Science, Kahramanmaraş Sütçü İmam University, Kahramanmaraş, Turkey

**Keywords:** Mouthguards, Sports dentistry, Traumatic dental injuries

## Abstract

**Objectives:**

Traumatic dental injury occurs during sports competitions, training, and practice and can be prevented by the use of mouthguards. For this reason, this study aimed to evaluate the knowledge and attitudes of sports science faculty students, physical education teachers, and athletes about mouthguards.

**Methods:**

Five hundred thirty-two participants were reached via social media. In the questionnaire consisting of 20 questions, questions about the sociodemographic characteristics of the participants were asked in part 1, and questions about their level of knowledge and attitudes about the mouthguards were asked in part 2. Descriptive statistical analysis and a chi-square test were used to evaluate the data.

**Results:**

The number of people who think that mouthguards protect the athlete from traumatic dental injury is 381 (71.6%). The number of people who think that mouthguards affect the performance of the athlete is positively 228 (42.9%). To protect against traumatic dental injury, 51.48% of the participants preferred the custom-made; 39.3% of them preferred the boil-bite; 33.22% of them preferred the standard/stock type mouth guard; and 22.96% of them preferred the helmet, and 18.26% of them preferred the face mask.

**Conclusions:**

The knowledge and attitude of sports science faculty students, physical education teachers, and athletes are low about mouthguards. Since these people who are engaged in sports have a high exposure to traumatic dental injuries, education should be given to increase their awareness about mouthguards.

## Introduction

Physical activities and sports contribute to the physical and mental positive developments of the individual [1]. However, retrospective reviews have reported that traumatic dental injury can be seen in almost one out of every 5 children between the ages of 11–13 in sports activities [2]. It was concluded that more than half of these injuries were seen during the competition and the rest during training [3]. According to the Federation Dentaire International classification, American football, ice hockey, hockey, martial arts, ice skating, skateboarding, lacrosse, rugby, and mountain biking are at high risk in terms of dental trauma, while basketball, socccer, handball, water polo, squash, gymnastics, diving, and, parachuting are at moderate risk [4]. In the meta-analysis including all sports injuries, the sports with the most dentofacial injuries were reported as rugby, basketball, handball, field hockey, and soccer [5].

Since these injuries cause aesthetic, functional, physical, economic, psychological, and social problems in the individual, the use of mouthguards during sports activities becomes meaningful [6]. Dentofacial injury is prevented by absorbing the energy of the incoming impact and reducing the force on the dental hard tissues [7, 8], mandibular condyle, and articular disc [9, 10]. Positive results of using a mouthguard on protection and performance have been seen in many studies [3, 11]. Contact athletes are advised to use safety equipment such as mouthguards to minimize impacts [12]. In general, there are 3 types of mouthguards: standard/stock, boil-bite (mouth-formed), and specially prepared by the dentist (custom-made). Properly fitted mouthguards absorb the high energy from traumatic blows, preventing the transfer of energy directly to the underlying teeth [7]. It has been reported that improper use of standard/stock and boil-bite may affect some problems such as discomfort, speech, and breathing problems [13, 14] and adversely affect exercise [15, 16]. On the other hand, it has been reported that a mouthguard specially prepared by a well-adapted dentist does not impair general sports activities and/or performance [15, 17, 18], has a negligible effect on cardiorespiratory endurance, and does not impair respiratory function [19–21], does not affect speech, does not cause nausea and has a longer service life [22, 23]. In the literature, there are some studies which are about the poor level of awareness for the prevention and emergency management of traumatic dental injuries and the use of mouthguards [24–26]. Accordingly, this study aims to evaluate the knowledge and attitudes of sports science faculty students, physical education teachers, and athletes about mouthguards.

## Methods

The study ethics committee was taken from the Kahramanmaraş Sütçü İmam University’s Non-Interventional Clinical Research Ethics Committee (2021/14).

A total of 532 participants, including sports science faculty students, physical education teachers, and athletes, were included in the study via Google form. The questionnaires of that study were developed from former articles [14, 27, 28]. The study has a total of 20 questions, and there are total of 7 questions (1–7), including age, gender, educational status, sports branch they are interested in, their position and status in sports, and professional experience in the first part. In the second part, there are a total of 13 questions (8–20) about mouthguard and sports dentistry. The responses to questions are based on multiple-choice and ‘yes/no/dont’ know’. After answering the questions, the authors which are one pediatric dentist and two sports scientists determined the ingredient validity of the questionnaire. The reliability of the questionnaire was evaluated with a test–retest approach to a total of 15 participants, 5 participants from each group, with an interval of 10 days. Subsequently, these participants were not included in the work. The kappa coefficient was between 0.78 to 0.83 for several questions indicating a good test–retest reliability [29].

For the calculation of the statistical analysis, the Jamovi statistical program (Version: 2.3.28) was used. The frequency of the participants according to demographic characteristics was calculated, and descriptive statistics were made. The answers to the questions about trauma were examined by chi-square analysis according to age, gender, and education level. Significance was set as *p* < 0.05.

## Results

Five hundred thirty-two people, including 160 females (30%) and 372 males (70%), participated in the study. 26% of the participants were 18–20; 53% of them were 21–30; 12% of them were 31–40; and 8.8% of them were 40 years of age or older. Of the participants, 306 (58%) were sports science faculty students; 142 (27%) were physical education teachers; 84 (16%) were athletes. The sports branches that the participants are interested in are respectively as follows: 141 (27%) football, 66 (12%) basketball, 64 (12%) volleyball, 58 (11%) wrestling, 49 (9.2%) swimming, 41 (7.7%) athletics, 28 (5.3%) boxing, 20 (3.8%) taekwondo, 15 (2.8%) handball and none, 14 (2.6%) skiing, 11 (2.1%) amateur kickboxing, 4 (0.8%) ice hockey, 1 (0.2%) water polo. Of the participants, 381 (72%) were amateurs, and 151 (28%) were professionals. Participants with 1–5 years of professional experience are 166 (31%); those with 6–10 years are 164 (31%), and those with more than 10 years are 202 (38%) (Table 1).

**Table 1:** Species of sports and years of knowledge of study participants

**Demographic characteristics**	*N* = 532
Age	
18–20	139 (26%)
21–30	282 (53%)
31–40	64 (12%)
40 and older	47 (8.8%)
Gender	
Male	372 (70%)
Female	160 (30%)
Education	
Student	306 (58%)
Athlete	84 (16%)
Physical Education Teacher	142 (27%)
Sports branch to be interested	
Wrestling	58 (11%)
Amateur kickboxing	11 (2.1%)
Box	28 (5.3%)
Karate	5 (0.9%)
Athletics	41 (7.7%)
Basketball	66 (12%)
Ice hockey	4 (0.8%)
Handball	15 (2.8%)
Volleyball	64 (12%)
Swimming	49 (9.2%)
Taekwondo	20 (3.8%)
Skiing	14 (2.6%)
Football	141 (27%)
Water polo	1 (0.2%)
None	15 (2.8%)
Position in the sport branch	
Amateur	381 (72%)
Professional	151 (28%)
National level in the sport branch	
Yes	88 (17%)
No	444 (83%)
Time in the sport branch	
More than 10 years	202 (38%)
6–10 years	164 (31%)
1–5 years	166 (31%)

There were 137 (25.8%) people with a previous history of trauma in sports dentistry. While there was a statistically significant difference between gender and trauma history (*p* = 0.037), there was no statistically significant difference between age and education levels (*p* = 0.093 and 0.104) (Table 2). The number of people who think that the mouthguards affect the performance of the athlete is 228 (42.9%) positively. While there was a statistically significant difference between gender and the belief that the mouthguards affected the athlete’s performance positively (*p* = 0.023), there was no statistically significant difference between age and education levels (*p* = 0.083 and 0.319) (Table 2). The number of people who previously knew about mouthguards was 156 (29.3%). The number of people who like to have more information about mouthguards is 354 (66.5%). While a statistically significant difference was observed in terms of age and gender in the respondents to this question (*p* = 0.011 and 0.046), there was no significant difference in terms of the education levels of the participants (*p* = 0.124) (Table 2). In avulsion injury, 200 people (37.6%) preferred treatment in the emergency department, 308 people (57.9%) in the dentist, and 24 (4.5%) at the scene. While there was a statistically significant difference in terms of age and treatment of avulsion injury (*p* = 0.013), there was no difference in terms of gender and education level (*p* = 0.59 and 0.073) (Table 2).

**Table 2:** Participants’ answers to the questionnaire on attitude of the mouthguards

Questions	Answers	**Gender**			***p*-value**	**Age**					***p*-value**		**Education Level**			***p*-value**
		**Male**	**Female**	**Total**		**18–20**	**21–30**	**31–40**	**40 -**	**Total**		**Students**	**Atlhetes**	**Teachers**	**Total**	
Previous history of trauma	Yes	107 (28.8%)	30(18.8%)	137 (25.8%)	0.037*	33 (23.7%)	68 (24.1%)	16 (25%)	20 (42.6%)	137 (25.8%)	0.093	67 (21.9%)	26 (31%)	44 (31%)	137 (25.8%)	0.104
	No	252 (67.7%)	121 (75.6%)	373 (70.1%)		100 (71.9%)	199 (70.6%)	47 (73.4%)	27 (57.4%)	373 (70.1%)		225 (73.5%)	53 (63.1%)	95 (66.9%)	373 (70.1%)	
	Don’t know	13 (3.5%)	9 (5.6%)	22 (4.1%)		6 (4.3%)	15 (5.3%)	1 (1.6%)	0 (0.0%)	22 (4.1%)		14 (4.6%)	5 (6%)	3 (2.1%)	22 (4.1%)	
Do you think that the mouthguards protect the athlete from traumatic injury?	Yes	266 (71.5%)	115 (71.9%)	381 (71.6%)	0.378	103 (74.1%)	197 (69.9%)	41 (64.1%)	40 (85.1%)	381 (71.6%)	0.241	212 (69.3%)	61 (72.6%)	108 (76.1%)	381 (71.6%)	0.414
	No	33 (8.9%)	9 (5.6%)	42 (7.9%)		11 (7.9%)	25 (8.9%)	5 (7.8%)	1 (2.1%)	42 (7.9%)		24 (7.8%)	9 (10.7%)	9 (6.3%)	42 (7.9%)	
	Don’t know	73 (19.6%)	36 (22.5%)	109 (20.5%)		25 (18.0%)	60 (21.3%)	18 (28.1%)	6 (12.8%)	109 (20.5%)		70 (22.9%)	14 (16.7%)	25 (17.6%)	109 (20.5%)	
Do you think that the mouthguards affects the performance of the athlete positively?	Yes	169 (45.4%)	59 (36.9%)	228 (42.9%)	0.023*	59 (42.4%)	113 (40.1%)	34 (53.1%)	22 (46.8%)	228 (42.9%)	0.083	128 (41.8%)	35 (41.7%)	65 (45.8%)	228 (42.9%)	0.319
	No	105 (28.2%)	40 (25%)	145 (27.3%)		35 (25.2%)	81 (28.7%)	11 (17.2%)	18 (38.3%)	14 (26.3%)		89 (29.1%)	17 (20.2%)	39 (27.5%)	145 (27.3%)	
	Don’t know	98 (26.3%)	61 (38.1%)	159 (29.9%)		45 (32.4%)	88 (31.2%)	19 (29.7%)	7 (14.9%)	159 (29.9%)		89 (29.1%)	32 (38.1%)	38 (26.8%)	159 (29.9%)	
Do you use mouthguard?	Yes	50 (13.4%)	29 (18.1%)	79 (14.8%)	0.363	21 (15.1%)	50 (17.7%)	6 (9.4%)	2 (4.3%)	79 (14.8%)	0.085	47 (15.4%)	13 (15.5%)	19 (13.4%)	79 (14.8%)	0.729
	No	316 (84.9%)	128 (80%)	444 (83.5%)		116 (83.5%)	225 (79.8%)	58 (90.6%)	45 (95.7%)	444 (83.5%)		253 (82.7%)	71 (84.5%)	120 (84.5%)	444 (83.5%)	
	Don’t know	6 (1.6%)	3 (1.9%)	9 (1.7%)		2 (1.4%)	7 (2.5%)	0 (0%)	0 (0%)	9 (1.7%)		6 (2%)	0 (0%)	3 (2.1%)	9 (1.7%)	
Do you know about mouthguards previously?	Yes	108 (29%)	48 (30%)	156 (29.3%)	0.770	38 (27.3%)	93 (33%)	15 (23.4%)	10 (21.3%)	156 (29.3%)	0.542	84 (27.5%)	27 (32.1%)	45 (31.7%)	156 (29.3%)	0.855
	No	254 (68.3%)	106 (66.2%)	360 (67.7%)		96 (69.1%)	182 (64.5%)	47 (73.4%)	35 (74.5%)	360 (67.7%)		212 (69.3%)	55 (65.5%)	93 (65.5%)	360 (67.7%)	
	Don’t know	10 (2.7%)	6 (3.8%)	16 (3%)		5 (3.6%)	7 (2.5%)	2 (3.1%)	2 (4.3%)	16 (3%)		10 (3.3%)	2 (2.4%)	4 (2.8%)	16 (3%)	
Do you like to have more about mouthguards?	Yes	245 (65.9%)	109 (68.1%)	354 (66.5%)	0.046*	82 (59%)	194 (68.8%)	38 (59.4%)	40 (85.1%)	354 (66.5%)	0.011*	206 (67.3%)	48 (57.1%)	100 (70.4%)	354 (66.5%)	0.124
	No	85 (22.8%)	24 (15%)	109 (20.5%)		39 (28.1%)	54 (19.1%)	13 (20.3%)	3 (6.4%)	109 (20.5%)		64 (20.9%)	24 (28.6%)	21 (14.8%)	109 (20.5%)	
	Don’t know	42 (11.3%)	27 (16.9%)	69 (13%)		18 (12.9%)	34 (12.1%)	13 (20.3%)	4 (8.5%)	69 (13%)		36 (11.8%)	12 (14.3%)	21 (14.8%)	69 (13%)	
Which option you will apply in the case the tooth is avülsed?	Emergency department	135 (36.3%)	65 (40.6%)	200 (37.6%)	0.59	65 (46.8%)	106 (37.6%)	21 (32.8%)	8 (17%)	200 (37.6%)	0.013*	114 (37.3%)	42 (50%)	44 (31%)	200 (37.6%)	0.073
	Dentist	219 (58.9%)	89 (55.6%)	308 (57.9%)		67 (48.2%)	163 (57.8%)	42 (65.6%)	36 (76.6%)	308 (57.9%)		177 (57.8%)	40 (47.6%)	91 (64.1%)	308 (57.9%)	
	Scene	18 (4.8%)	6 (3.8%)	24 (4.5%)		7 (5%)	13 (4.6%)	1 (1.6%)	3 (6.4%)	24 (4.5%)		15 (4.9%)	2 (2.4%)	7 (4.9%)	24 (4.5%)	
Which condition do you prefer if the tooth is avülsed?	Alcohol	52 (14%)	14 (8.8%)	66 (12.4%)	0.253	13 (9.4%)	38 (13.5%)	10 (15.6%)	5 (10.6%)	66 (12.4%)	0.214	35 (11.4%)	10 (11.9%)	21 (14.8%)	66 (12.4%)	0.271
	In the mouth	23 (6.2%)	7 (4.4%)	30 (5.6%)		8 (5.8%)	13 (4.6%)	4 (6.2%)	5 (10.6%)	30 (5.6%)		17 (5.6%)	3 (3.6%)	10 (7%)	30 (5.6%)	
	Toothbrush	4 (1.1%)	4 (2.5%)	8 (1.5%)		5 (3.6%)	3 (1.1%)	0 (0%)	0 (0%)	8 (1.5%)		5 (1.6%)	3 (3.6%)	0 (0%)	8 (1.5%)	
	Napkin	128 (34.4%)	51 (31.9%)	179 (33.6%)		54 (38.8%)	92 (32.6%)	18 (28.1%)	15 (31.9%)	179 (33.6%)		111 (36.3%)	29 (34.5%)	39 (27.5%)	179 (33.6%)	
	Water	135 (36.3%)	67 (41.9%)	202 (38%)		42 (30.2%)	115 (40.8%)	28 (43.8%)	17 (36.2%)	202 (38%)		107 (35%)	34 (40.5%)	61 (43%)	202 (38%)	
	Milk	30 (8.1%)	17 (10.6%)	47 (8.8%)		17 (12.2%)	21 (7.4%)	4 (6.2%)	5 (10.6%)	47 (8.8%)		31 (10.1%)	5 (6%)	11 (7.7%)	47 (8.8%)	

*p<0.05

**Fig. 1:**
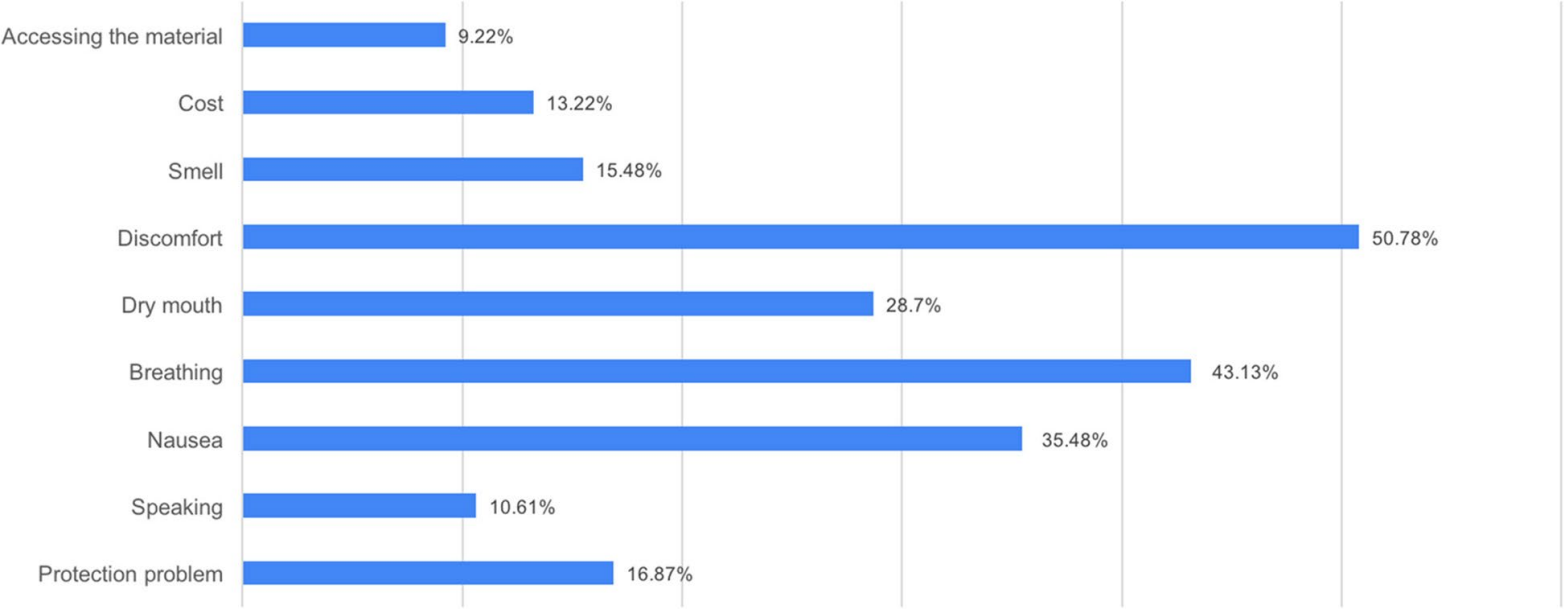
Disadvantages of the mouthguards

Participants listed the disadvantages of mouthguarding with multiple responses, to which they may respond more than once, as follows: 50.78% of participants reported discomfort; 43.13% reported breathing problems, 35.48% reported nausea; 28.7% reported dry mouth; 16.87% reported not providing adequate protection; 15.48% reported smell; 13.22% reported cost; 10.61% reported speaking; 9.22% reported problems accessing the material (Fig. 1).

**Fig. 2:**
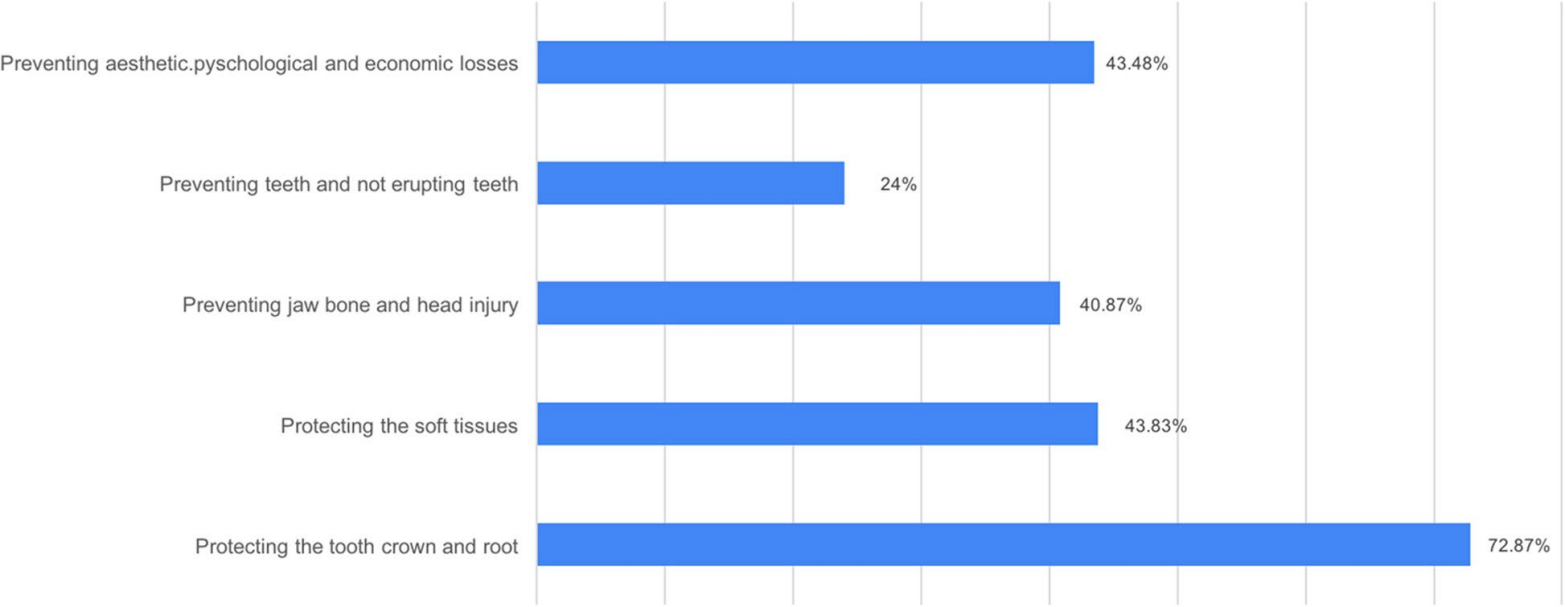
Advantages of the mouthguards

Participants discussed the advantages of the mouthguards with multiple responses respectively: 72.87% of them reported protecting the tooth crown and root, 43.83% of them reported protecting soft tissues such as lips and tongue, 43.48% of them reported preventing aesthetic, psychological, and economic losses, 40.87% of them reported preventing jaw bone and head injury, and 24% of them reported preventing teeth that had not erupted or were in progress (Fig. 2).

**Fig. 3:**
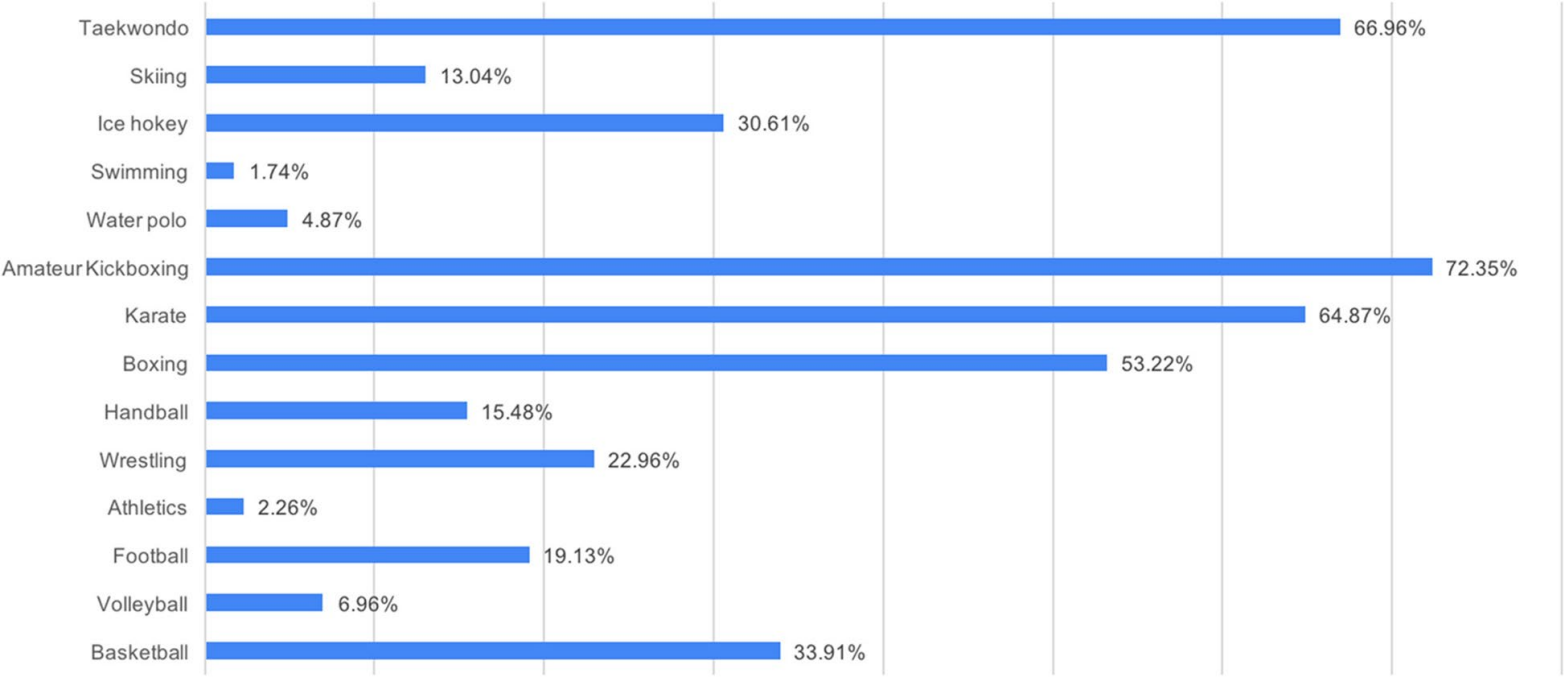
Do you think that which sports require using the mouthguards?

Participants responded to the question about the sport branch that requires the need for mouthguards, to which they can give more than one response, as follows; 72.35% of them as amateur kickboxing, 66.96% of them as taekwondo, 64.87% of them as karate, 53.22% of them as boxing, 33.91% of them as basketball, 30.61% of them as ice hockey, 22.96% of them as wrestling, 19.13% of them as football, 15.48% of them as handball, 13.04% of them as skiing, 6.96% of them as volleyball, 4.87% of them as water polo, 2.26% of them as athletics, and 1.74% of them swimming (Fig. 3).

**Fig. 4:**
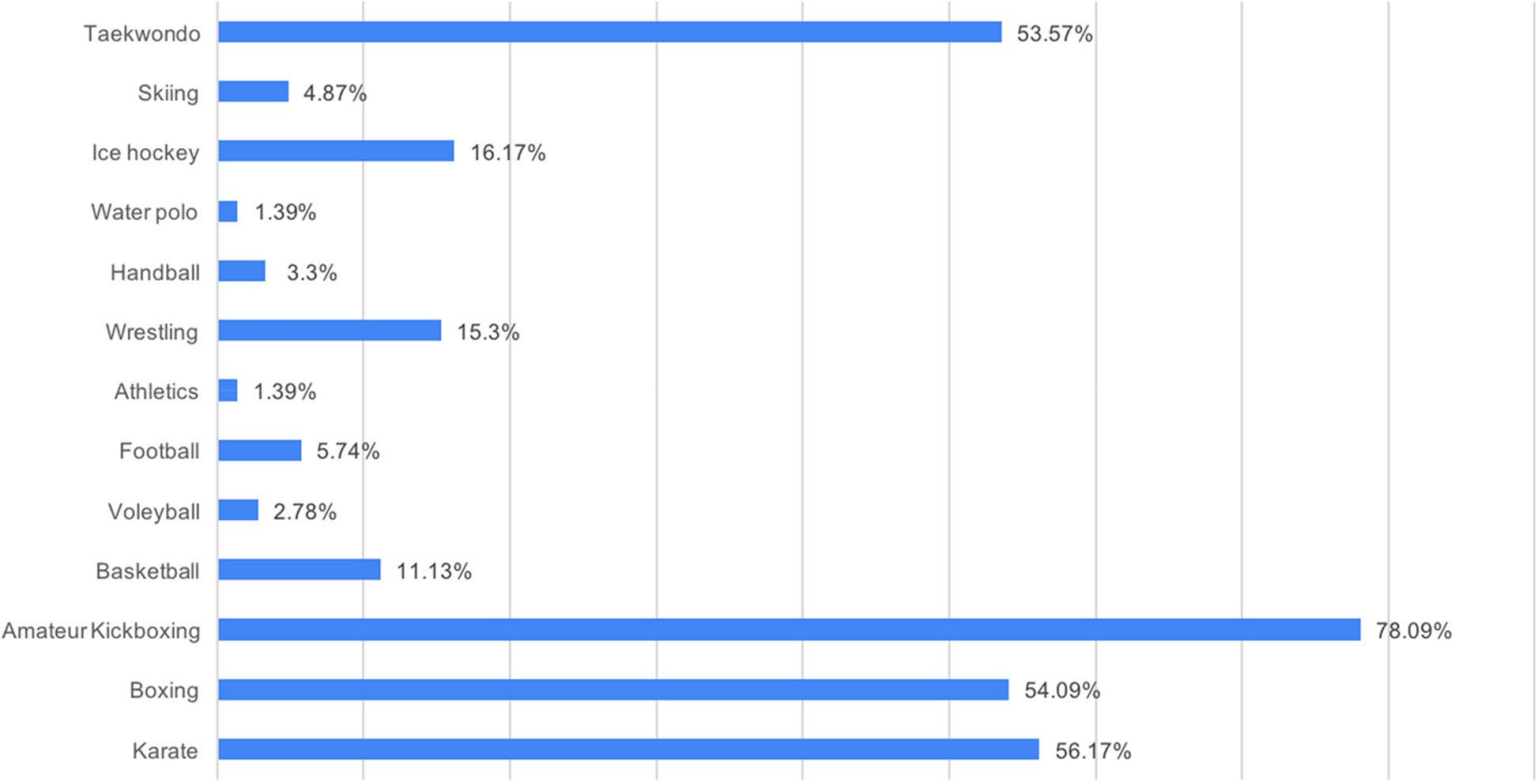
Do you think that which sports have obligation about using mouthguards in our country?

Participants listed the branches with the obligation to use mouthguards, to which they can give more than one response, as follows; 78.09% of them as amateur kickboxing, 56.17% as karate, 54.09% as boxing, 53.57% as taekwondo, 16.17% as ice hockey, 15.3% as wrestling, 11.13% as basketball, 5.74% as football, 4.87% as skiing, 3.3% as handball, 2.78% as volleyball, 1.39% as athletics and water polo (Fig. 4).

**Fig. 5:**
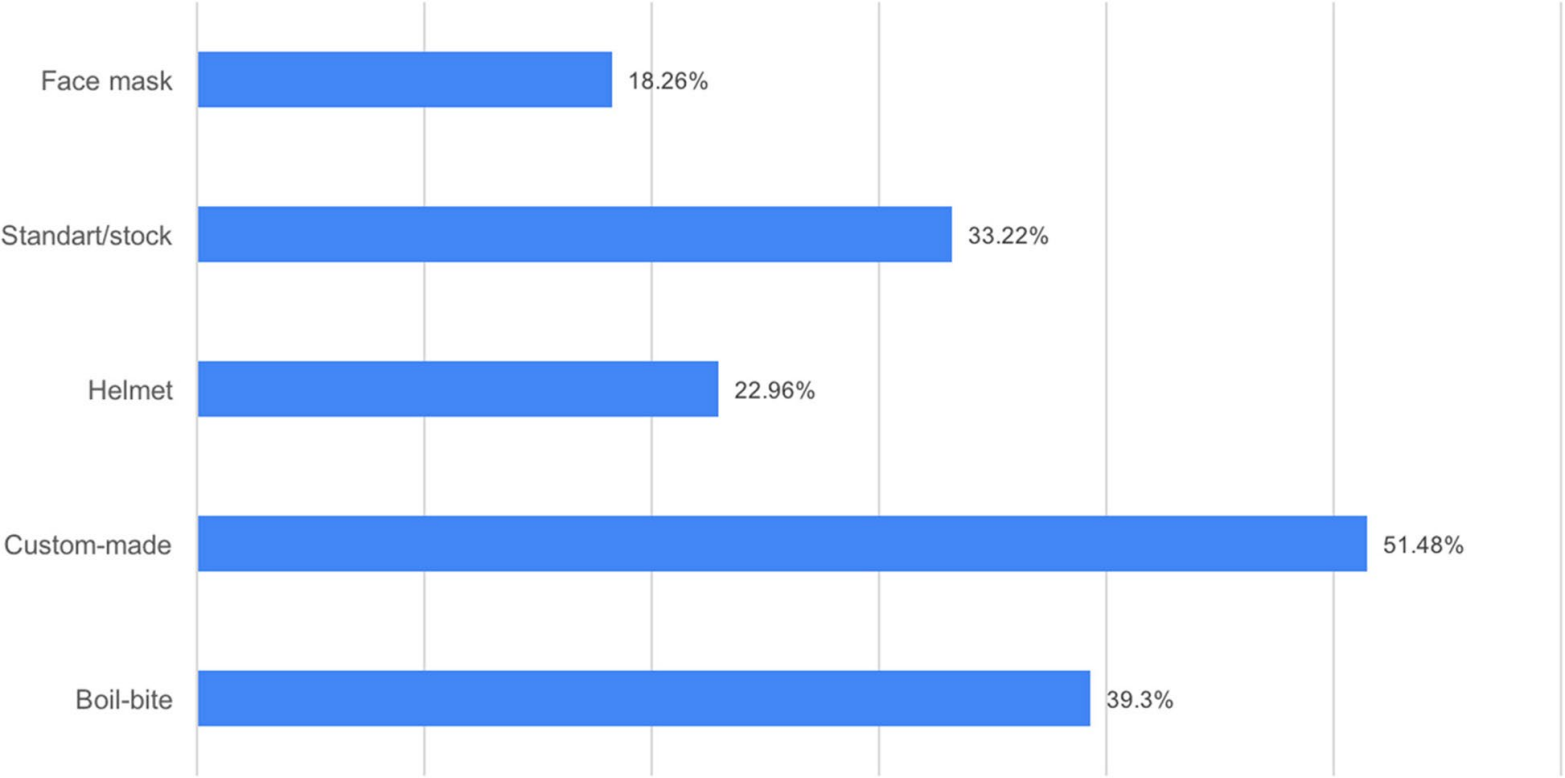
Dou you think that which method may be used to protect against traumatic dental injury?

To protect against traumatic dental injury, 51.48% of the participants preferred the custom-made; 39.3% of them preferred the boil-bite; 33.22% of them preferred the standard /stock type mouthguard; and 22.96% of them preferred the helmet; and 18.26% of them preferred the face mask (Fig. 5).

## Discussion

Athletes, coaches, athletic directors, families, doctors and dentists should be aware of the risk, history, and treatment of dental injuries in individuals who play, train, and practice sports. These individuals have a good command of the type and treatment of orofacial injuries treat the athlete during the competition and contribute to the continuity of the game [30]. Thus, the physical and psychological damage that will be created in the athlete is minimized [31]. This study aimed to measure the awareness of sports science faculty students, physical education teachers, and athletes about mouthguards.

The American Academy of Pediatric Dentistry (AAPD) states that falls, bumps, hard surfaces, and contact with sports-related equipment pose a risk of orofacial injury in all sports activities. Most of these injuries are prevented by the use of mouthguards, face masks, and helmets [32].

When the studies in the literature are examined, the participants with a previous history of trauma vary between 17.5-83% [14, 33–35], while, in this study, this rate is low (25.8%). Similar to this study, this rate is 22.3% in the study conducted by Tulunoğlu and Özbek [36]. In this study, male exposed to trauma in a similar way to a study by Özbay et al. [37] were statistically significantly higher than female (*p* = 0.037) (Table 2). This result may be related to the fact that male reflects their strong physical structure to the sports competition. In a study, although it was reported that males had more injuries than females, it was found that injuries in females resulted in more surgeries [38]. On the other hand, Traebert et al. [39] argued that females have similar risk factors for orofacial injuries as male. Another interesting result found in this study is that although there is no statistically significant difference between age and trauma history (*p* = 0.093) (Table 2), similar to Tulunoglu and Ozbek [36]’s study, the trauma history of the participants increases as they get older. The history of trauma may have also increased, possibly as the number of sports competitions experienced increased as individuals got older. On the other hand, in the study conducted by Esmaeilpoor et al. [14], it was reported that young athletes had a higher history of trauma than older people. Also, sports-related orofacial injury is associated with multiple components like the kind of sport, geographical location, specimen size, age, level of match, requirements for the use of safety equipment, and duration of exposure [4, 40, 41].

In the study by Sepet et al. [33], Galic et al. [42] and Elareibi et al. [25] 55.4%, 97.3% and 89.4% of the participants knew that they should use mouthguards, respectively; only 11.2% of participants, 41% and 14.8% of them used mouthguards. Similarly, in our study, although most of the participants knew that they should use mouthguards (71.6%), very few chose to use them (14.8%) (Table 2). When the studies conducted in Turkey are examined, the rate of mouthguards use is 0–55.8% [27, 36, 37]. On the other hand, the study conducted by Vidovic-Stesevic et al. [43] included 420 athletes, and 98% reported using mouthguards.

Protective equipment provided to children interested in football, lacrosse, and ice hockey has been observed to significantly reduce dental and facial injuries. Sports such as baseball, basketball, football, softball, wrestling, volleyball, and gymnastics are insufficient to protect boys and girls from injury. Young people who participate in free activities such as skateboarding, skating, and cycling use more protective equipment [44–46]. In this study, in the multiple-choice question related to sports requiring the need for mouthguards, the most answers were given to close contact sports such as 72.35% amateur kickboxing, 66.96% taekwondo, 64.87% karate, and 53.22% boxing respectively, and the least responses were given to sports such as 1.74% swimming, 2.26% athletics and 4.87% water polo (Fig. 3). Differences in the rates of mouthguards use may be related to the study group’s age, education level, and the sport they are interested in (team sport or individual sport).

According to an interesting result found in this study, 51.48% of the participants preferred the custom-made, 39.3% preferred the boil-bite, 33.22% preferred the standard/stock type mouth guard (Fig. 5). In some studies, in the literature, mouthguards shaped in the mouth were preferred [27, 36], while there were also participants who preferred the stock type [14]. In our study, the participants preferred custom-made mouthguards (51.48%). AAPD recommends custom-made mouthguards for all sports activities due to the risk of traumatic dental injury [32].

In this study, the participants stated the feeling of discomfort as the most common disadvantage of using mouthguards, which is a multiple-choice question (Fig. 1), and this result is consistent with the literature [47–49]. Nevertheless, since screaming in combat sports contributes to the physical and mental motivation of the athlete during the competition, the use of mouthguards can be considered a disadvantage [36]. In some studies, athletes have reported that stock or mouth-formed mouthguards cause breathing and speech problems or jaw and muscle fatigue, but this problem is solved by custom-made mouthguards that adapt well to the gums and teeth [48–52].

In the study conducted by Yeşil Duymuş and Gungor [28] and Cetınbas and Sönmez [27], 78% and 95.5% of the participants liked to have more about mouthguards; in this study, this rate was 66.5%. Since pediatric dentistry is the branch of dentistry that deals with children and adolescents with a higher frequency of trauma, it supports directing athletes and coaches to raise more awareness about mouthguards. The National Association of Athletics Coaches recommends that coaches be trained in the use of a properly placed mouthguard and that athletes should participate in activities associated with the risk of orofacial injury [53]. Thus, athletes will adopt the mouthguard and reflect it in routine use [24, 38]. Regarding the question of which branch should be used as mouthguard in Turkey, 78.09% of participants answered as kickboxing, 56.17% as karate, and 54.09% as boxing (Fig. 4). Similar to the General Directorate of Youth Sports [54], in this study, the necessity of using mouthguards in sports related to direct contact was more accepted. Due to the increasing number of athletes around the world, dental trauma is an important dental health problem. For this purpose, sports committees need to regulate the use of mouthguards as mandatory protective equipment. It makes sense for dentists to encourage the use of customizing mouthguards for athletes [24]. This protects the athlete from orofacial injury and contributes to the quality of life of the athlete due to its aesthetic, speech, economic and psychological effects.

## Limitations

In the study in question, the participants were asked questions in the form of Google surveys, and social networks such as WhatsApp, e-mail, Facebook, and Instagram were also tried to reach participants. Conducting a face-to-face survey can make participants more willing. In addition, this led to data loss, and some participants were excluded from the study because they did not respond to every question. The study data were obtained only from participants in Turkey, and the inclusion of international athletes may increase the scope of the study.

## Conclusion

The results of the study have revealed that there was not enough knowledge about the use of mouthguards. The use of mouthguards should not be left to personal preference, as it may make a difference in the quality of life of the athlete. For this purpose, the use of mouthguards should be mandatory in medium-risk sports as well as high-risk sports. To create oral protective awareness of physical education teachers and athletes against injuries in sports dentistry, it may be necessary to provide training on this subject in the faculty of sports science to put courses in the curriculum and to organize symposiums with posters and videos.

## Data Availability

The datasets used and/or analysed during the current study available from the corresponding author on reasonable request.

## Abbreviation

AAPD: American Academy of Paediatric Dentistry
